# Identification and validation of novel prognostic biomarkers and therapeutic targets for non-small cell lung cancer

**DOI:** 10.3389/fgene.2023.1139994

**Published:** 2023-03-16

**Authors:** Li-Ting Lai, Yuan-Hui Ren, Ya-Jun Huai, Yu Liu, Ying Liu, Shan-Shan Wang, Jin-Hong Mei

**Affiliations:** ^1^ Department of Oncology, The First Affiliated Hospital of Nanchang University, Nanchang, Jiangxi, China; ^2^ Department of Pathology, The First Affiliated Hospital of Nanchang University, Nanchang, Jiangxi, China; ^3^ Institute of Molecular Pathology, Nanchang University, Nanchang, Jiangxi, China

**Keywords:** non-small cell lung cancer, gene expression omnibus, differentially expressed genes, protein-protein interaction, biological process

## Abstract

**Background:** Despite the significant survival benefits of anti-PD-1/PD-L1 immunotherapy, non-small cell lung cancer (NSCLC) remains one of the most common tumors and major causes of cancer-related deaths worldwide. Thus, there is an urgent need to identify new therapeutic targets for this refractory disease.

**Methods:** In this study, microarray datasets GSE27262, GSE75037, GSE102287, and GSE21933 were integrated by Venn diagram. We performed functional clustering and pathway enrichment analyses using R. Through the STRING database and Cytoscape, we conducted protein-protein interaction (PPI) network analysis and identified the key genes, which were verified by the GEPIA2 and UALCAN portal. Validation of actin-binding protein anillin (ANLN) was performed by quantitative real-time polymerase chain reaction and Western blotting. Additionally, Kaplan-Meier methods were used to compute the survival analyses.

**Results:** In total, 126 differentially expressed genes were identified, which were enriched in mitotic nuclear division, mitotic cell cycle G2/M transition, vasculogenesis, spindle, and peroxisome proliferator-activated receptor signaling pathway. 12 central node genes were identified in the PPI network complex. The survival analysis revealed that high transcriptional levels were associated with inferior survival in NSCLC patients. The clinical implication of ANLN was further explored; its protein expression showed a gradually increasing trend from grade I to III.

**Conclusion:** These Key genes may be involved in the carcinogenesis and progression of NSCLC, which may serve as useful targets for NSCLC diagnosis and treatment.

## Introduction

Lung cancer is the most common cause of cancer-related death worldwide, wherein NSCLC accounts for 85% of lung cancer cases (Sung et al., 2021). An increased understanding of the biology and pathogenic genomic changes in NSCLC has led to advances and developments in its treatment. Particularly, the emergence of molecularly targeted therapies and immunotherapy has fundamentally changed the way NSCLC patients are treated (Jordan et al., 2017). A large number of genes have been recognized as drug targets and their molecular alterations, including epidermal growth factor receptor mutations, proto-oncogene receptor tyrosine kinase 1 rearrangements, anaplastic lymphoma kinase rearrangements, and BRAF V600E mutations, could predict the response to treatment (Stella et al., 2013). Testing for these genes is becoming increasingly routine and has yielded motivating results.

However, the incidence of rearrangement, fusion, or over-expression of these genes in NSCLC patients are very low, leading to limited availability of molecular targeted therapies for these genes. For example, aberrantly activations of ALK was found in approximately 4% of NSCLC tumors, and chromosomal rearrangement of ROS1 has been identified in approximately 1% of NSCLC patients (Wong et al., 2009; Gainor and Shaw, 2013). EGFR somatic activating mutations were found in approximately 20% of advanced NSCLC patients, and represented a paradigm for the use of tyrosine kinase inhibitors for subsets of cancer treatment. However, acquired resistance inevitably occurs in these cases (Yu et al., 2015). In addition, there is currently a very limited number of drug targets for other subtypes of lung cancer, such as squamous cell and large cell carcinoma, other than adenocarcinoma. Furthermore, targeted drugs developed for lung adenocarcinoma are basically ineffective for lung squamous cell carcinoma (Rekhtman et al., 2012). As for immunotherapy, the improvement in survival of lung cancer patients by blocking the immune checkpoint PD-1/PD-L1 is encouraging. However, only about 20% of patients benefit, and resistance is likely to develop after the initial response (Topalian et al., 2019). Thus, identifying potential gene targets or pathway alterations in this refractory disease is urgently needed.

Currently, the availability of information about the human genome and proteome, especially those that assist in the development of new anti-cancer agents, is largely dependent on advances in bioinformatics. As an enabling technology, bioinformatics bridges the gap between sequence information and clinical practice, and it has evolved into multiple ways to enable us not only to identify “driver” and “passenger” genes toward neoplasia, but also to comprehend genetic alterations and mechanisms in cancer (Mount and Pandey, 2005).

In this study, four microarrays, namely GSE27262, GSE75037, GSE102287, and GSE21933, were integrated and analyzed. Differentially expressed genes (DEGs) between NSCLC samples and corresponding normal specimens were analyzed. Subsequently, Gene Ontology (GO) and Kyoto Encyclopedia of Genes and Genomes (KEGG) pathway enrichment analyses of the DEGs were developed. The PPI network was developed by the Search Tool for the Retrieval of Interacting Genes (STRING) database. We screened out the key genes with the supreme connectivity in the network and evaluated their prognostic value, which would be helpful for further development of prognostic biomarkers and novel therapeutic targets for NSCLC patients.

## Materials and methods

### Microarray datasets information

The National Center for Biotechnology Information Gene Expression Omnibus (GEO) is an open-access database for data regarding next-generation sequencing, microarray, and other forms of high-throughput gene data (Barrett et al., 2013), from which the microarray datasets of lung cancer samples and adjacent non-malignant samples (GSE27262, GSE75037, GSE102287, and GSE21933) were downloaded. Gene expression profiles of GSE27262 and GSE102287 were based on platform GPL570 [HG-U133_Plus_2] Affymetrix Human Genome Array, with 25 lung adenocarcinoma tissues *versus* 25 adjacent normal specimens and 32 NSCLC samples *versus* 34 normal samples, respectively. GSE75037 was based on platform GPL6884 HumanWG-6 v3.0 expression beadchip, including 83 adenocarcinomas and 83 adjacent normal samples. GSE21933 was based on platform GPL6254 Phalanx Human OneArray, including 21 NSCLC tissues and 21 matched adjacent non-malignant tissues.

### Data analysis

GEO2R, a network application based on R that utilizes the Bioconductor (R packages) to analyze GEO data, was used to identify DEGs between lung cancer and adjacent non-malignant specimens. The selection criteria, |logFC| > 2.0, and adjusted *p* < 0.05 were used to define the DEGs. We analyzed each dataset and intersected them using Venn diagrams.

### GO and KEGG pathway enrichment analysis

The GO knowledgebase was composed of ontology and ontology annotations. As of 2018, there were approximately 45,000 terms in GO, including CC, BP, and MF terms (The Gene Ontology Consortium, 2017). R software version 4.0.3 (clusterProfiler and ggplot2 packages) was used for gene classification and GO, KEGG pathway enrichment analyses. Statistical significance was set at *p* < 0.05.

### PPI network visualization

STRING v11, an online resource with currently the largest number of proteins (24.6 million) and broad data sources (Szklarczyk et al., 2019), was employed to explore protein-protein associations among the DEGs. In addition, Cytoscape software was used for the visualization of the protein interaction network and the analyzation of the interaction of the candidate DEGs that encode proteins in NSCLC. The top 12 molecules with the strongest connectivity in the network were identified as key genes by CytoHubba, a plug-in of Cytoscape.

### Genetic alteration analysis and enrichment analysis of the key gene-related drugs

Through the data of lung adenocarcinoma and lung squamous cell carcinoma of the TCGA project and the Sangerbox platform, we obtained the mutation profile of 12 key genes in NSCLC. Furthermore, we have enriched and analyzed these key gene-related drugs by using Enrichr platforms (https://maayanlab.cloud/Enrichr/). We used Diseases/Drugs and DSigDB module for cluster analysis.

### Survival analysis

To evaluate the effect of the 12 key genes on prognosis of NSCLC patients, we used the Kaplan–Meier plotter (http://kmplot.com/analysis/), an interactive database for validation of prognostic biomarkers that contains mRNA, miRNA, protein data, and clinical information from a variety of cancer patients. Patients with NSCLC were grouped based on their mRNA levels and hazard ratios, and the respective *p* values were calculated. In addition, we verified the survival analysis using the TCGA database (https://portal.gdc.cancer.gov). We downloaded and collated lung adenocarcinoma and squamous cell carcinoma RNAseq data and clinical data from the TCGA database; Survival package of R software was used to test the proportional risk hypothesis, and the results were visualized using survminer package and ggplot2 package.

### Expression analysis and clinicopathological association

The expression validation of the key genes was performed based on RNA sequencing data produced by The Cancer Genome Atlas (TCGA) and Genotype-Tissue Expression (GTEx) project. Tissue-wise expression analyses of key genes between 969 NSCLC samples and 685 non-malignant samples from TCGA and the GTEx project were profiled using GEPIA2 (http://gepia2.cancer-pku.cn/).

Clinicopathologic features of patients with NSCLC, including pathologic stage, tumor grade, age, gender, living status, and body weight, were obtained from the Clinical Proteomic Tumor Analysis Consortium (CPTAC) Confirmatory/Discovery dataset. Proteomic analyses of lung cancer and normal samples were performed using UALCAN, an open network repository for investigation on gene expression and its disease association (Chandrashekar et al., 2017). Furthermore, we obtained its immunohistochemical results from the HPA database (The Human Protein Atlas https://www.proteinatlas.org/).

### Cell culture

Lung cancer cell lines NCI-H1975, NCI-H1650, A549, and NCI-H1299, and normal lung epithelial cell line BEAS-2B were bought from the Shanghai Cell Bank and ICELL Company. Cells were cultured in Roswell Park Memorial Institute-1640 (RPMI-1640; Solarbio, Beijing, China) and Dulbecco’s modified Eagle’s medium (DMEM; Solarbio, Beijing, China) supplemented with 10% fetal bovine serum (FBS; Gemini, California, United States) and maintained at 37 °C thermostatic and humidified cell incubator with 5% CO2.

### RNA extraction and qRT-PCR

Total RNA was extracted from NSCLC cell lines and normal lung epithelial cell lines with an RNA extraction kit (Axygen; Silicon Valley, United Ststes) and reverse transcription was performed using a cDNA Synthesis Kit (Vazyme Biotech, Nanjing, China). Quantitative real-time polymerase chain reaction (qRT-PCR) was carried out using Bio-Rad CFX96 Touch with ChamQ SYBR^®^ Green qRT-PCR Master Mix. All qRT-PCRs were performed three times and measured using 2^−ΔΔCT^algorithm. Primer sequences were as follows: ANLN, Former: TCT​TCG​TGG​CCG​ATT​TGA​CA, Reverse: TGG​ACT​TAC​CAC​ACC​AAC​TGT; GAPDH, Former: CGA​GCC​ACA​TCG​CTC​AGA​CA, Reverse: GTG​GTG​AAG​ACG​CCA​GTG​GA.

### Western blotting

We extracted proteins for Western blotting using RIPA lysis buffer (Solarbio, Beijing, China; R0010) and phenylmethylsulfonyl fluoride protease inhibitors (Solarbio, Beijing, China; IP0280). The BCA Protein Assay Kit (Vazyme Biotech, Nanjing, China; E112-02) was used for protein concentration determination. The Western blot system was established using the Bio-Rad Bis-Tris Gel system according to the manufacturer’s instructions. Proteins isolated by SDS-PAGE were electroblotted onto polyvinylidene fluoride membranes and incubated with a primary antibody (dilution: 1:1250) overnight in a shaker at 4°C. They were then incubated in a shaker for 1 h with horseradish peroxidase labeled secondary antibody (dilution: 1:20000) at 25°C. After rinsing, a multi-functional chemiluminescent imaging system (Analytik-Jena, United States) was used for development.

## Results

### Screening of DEGs

**FIGURE 1:**
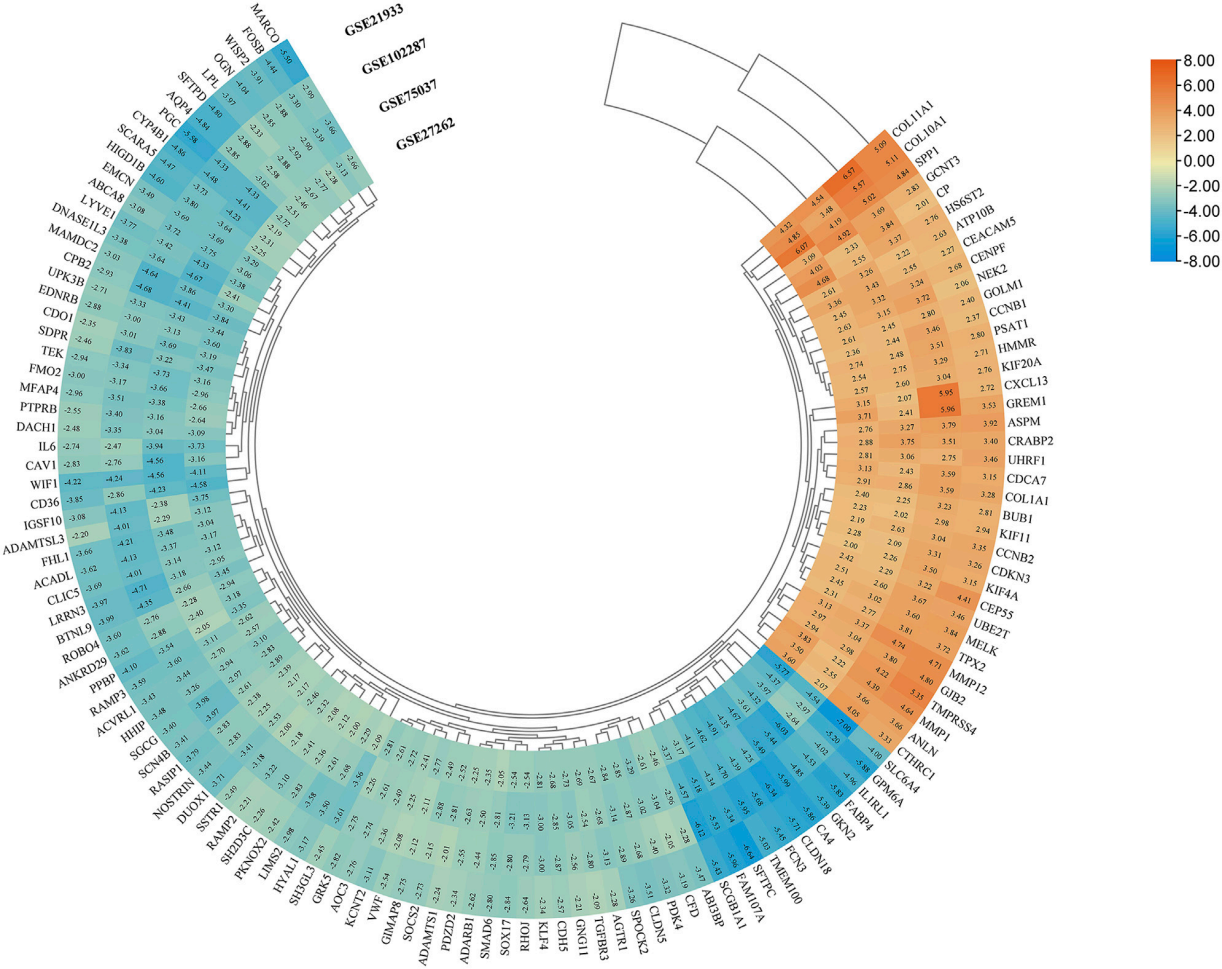
Screening of DEGs in four gene expression datasets (|logFC| > 2 and *p* < 0.05). Heatmap of all overlapping DEGs. Upregulated DEGs, orange; Downregulated DEGs, blue; logFC, log fold change; DEG, differentially expressed gene.

**FIGURE 2:**
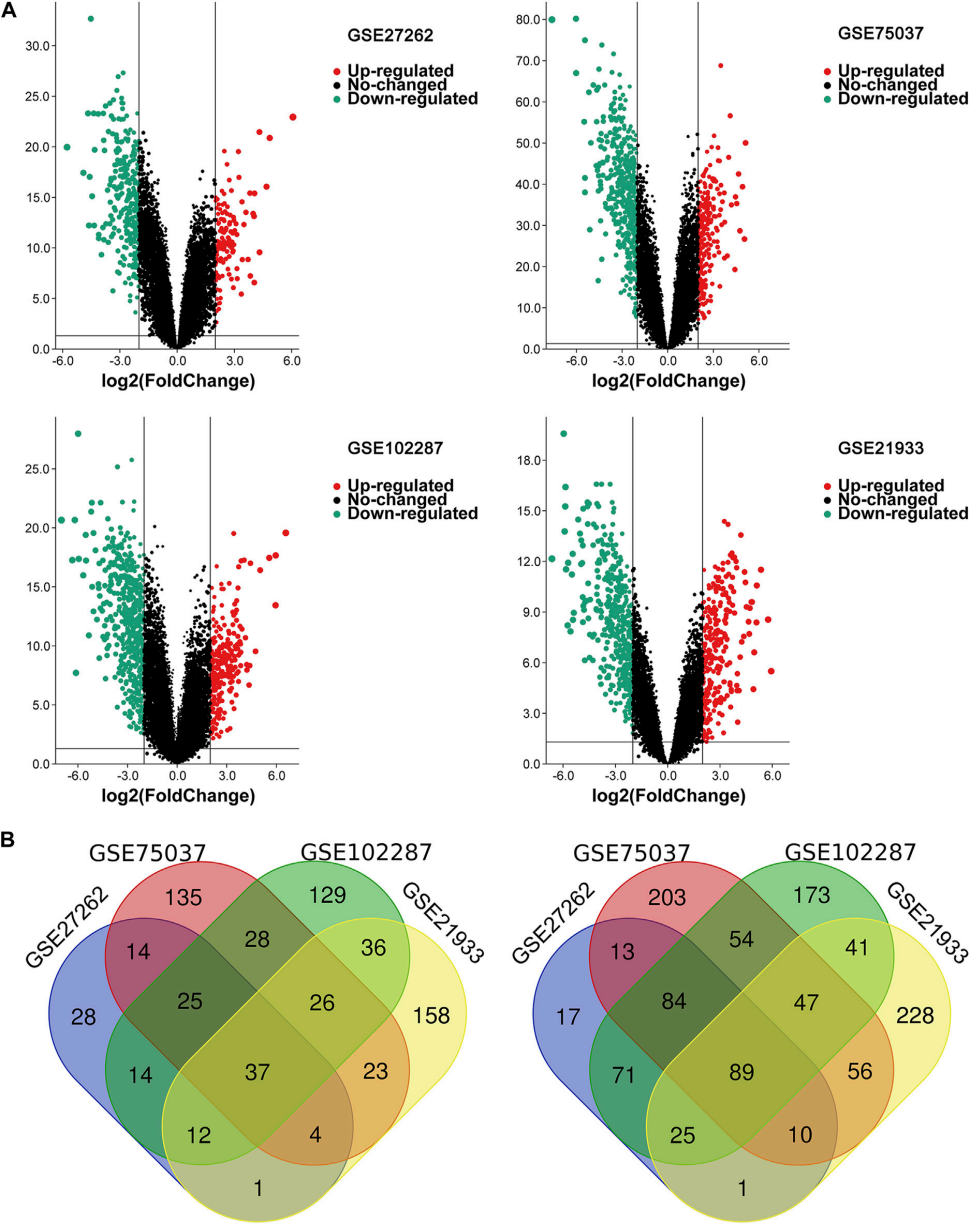
The overlapping DEGs of the four gene expression datasets. **(A)** Volcano plots of each gene expression profiles in NSCLC and normal tissues. **(B)** Venn diagrams of DEGs. The one on the left refers to 37 upregulated DEGs; The right one refers to 89 downregulated DEGs; NSCLC, non-small cell lung cancer; DEG, differentially expressed gene.

Four microarray datasets (GSE27262, GSE75037, GSE102287, and GSE21933) were selected in this study, and their statistics are shown in Table 1. Clustering of all overlapping DEGs is shown in the heatmap (Figure 1). In accordance with the selection criteria, |logFC| > 2.0 and adjusted *p* < 0.05, a total of 445, 845, 891, and 794 DEGs were identified from the GSE27262, GSE75037, GSE102287, and GSE21933 microarrays, respectively, as shown in the volcano plots (Figure 2A). After intersecting the DEGs of the four databases, 126 DEGs including 37 upregulated genes and 89 downregulated genes, were found to be significant in all four microarray datasets (Figure 2B).

**TABLE 1:** The composition of four different gene expression omnibus datasets.

GEO series	NSCLC	Normal	Total number
GSE75037	83	83	166
GSE21933	21	21	42
GSE27262	25	25	50
GSE102287	32	34	66

Abbreviations: NSCLC, non-small cell lung cancer; GEO, gene expression omnibus.

### Functional annotation and pathway enrichment analyses

**FIGURE 3:**
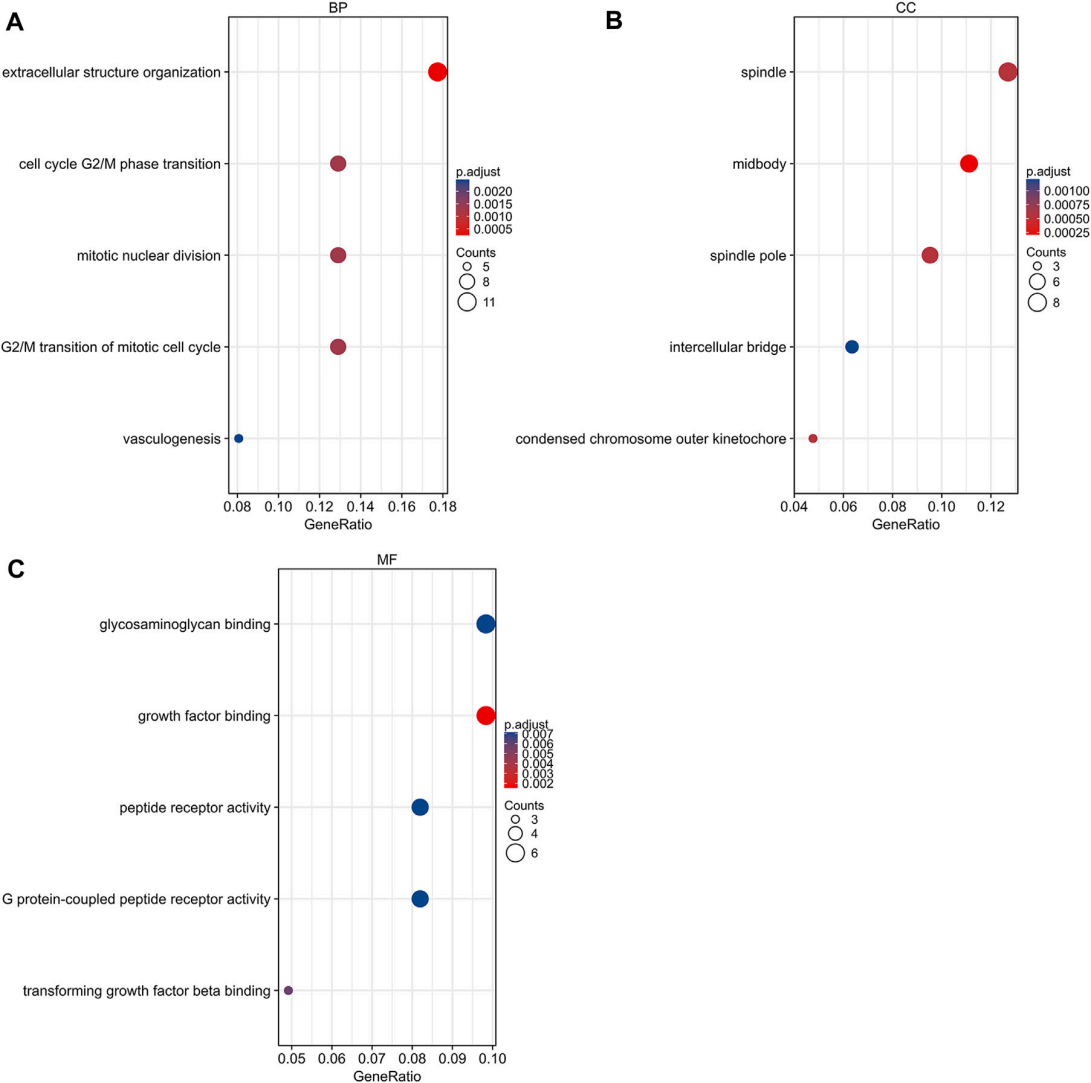
Gene ontology analysis of DEGs. **(A)** Biological process terms of DEGs. **(B)** Cellular component terms of DEGs. **(C)** Molecular function terms of DEGs. DEG, differentially expressed gene.

**FIGURE 4:**
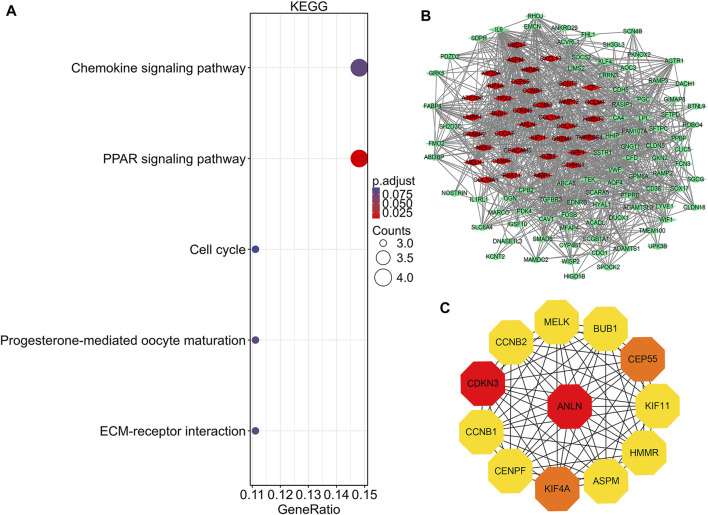
KEGG pathway analysis, protein-protein interaction network construction, and module analysis. **(A)** Significantly enriched KEGG pathway terms of DEGs in NSCLC. **(B)** DEGs protein–protein interaction network complex. Red nodes refer to upregulated genes. Green nodes refer to downregulated genes. Edges represent protein-protein associations. **(C)** Top 12 key genes with high connectivity in the network. The shade of the color indicates the strength of the connection. NSCLC, non-small cell lung cancer; DEG, differentially expressed gene.

GO and KEGG pathway analyses were conducted using R 4.0.3 (clusterProfiler, org.Hs.e.g.,.db, and ggplot2 packages). DEGs were basically enriched in mitotic nuclear division, cell cycle, G2/M phase transition, vasculogenesis, G2/M transition of mitotic cell cycle in biological process (BP) terms, spindle, midbody, condensed chromosome outer kinetochore in cellular components (CC) terms, and growth factor binding, G protein-coupled peptide receptor activity, and peptide receptor activity in molecular functions (MF) terms (Figure 3). The KEGG pathway analysis found that the DEGs were predominantly involved in the peroxisome proliferator-activated receptors (PPAR) signaling pathway, cell cycle, and ECM-receptor interaction pathway (Figure 4A).

### PPI network construction and key gene identification

There were 126 nodes and 1,054 edges in the PPI network with an enrichment *p*-value of <1.0e-16 (Figure 4B). Twelve central node genes, including ANLN, cyclin-dependent kinase inhibitor 3 (CDKN3), kinesin family member 4A (KIF4A), centrosomal protein 55 kDa (CEP55), G2/mitotic-specific cyclin-B1 (CCNB1), kinesin family member 11 (KIF11), G2/mitotic-specific cyclin-B2 (CCNB2), maternal embryonic leucine zipper kinase (MELK), hyaluronan-mediated motility receptor (HMMR), abnormal spindle-like microcephaly associated protein (ASPM), centromere protein F (CENPF), and checkpoint serine/threonine-protein kinase (BUB1), were identified among the 126 nodes by using CytoHubba of Cytoscape (Figure 4C). Furthermore, ANLN was the top gene in the network with the highest connectivity and maximum neighborhood component (Table S1).

### Transcriptional level validation of the 12 key genes

**FIGURE 5:**
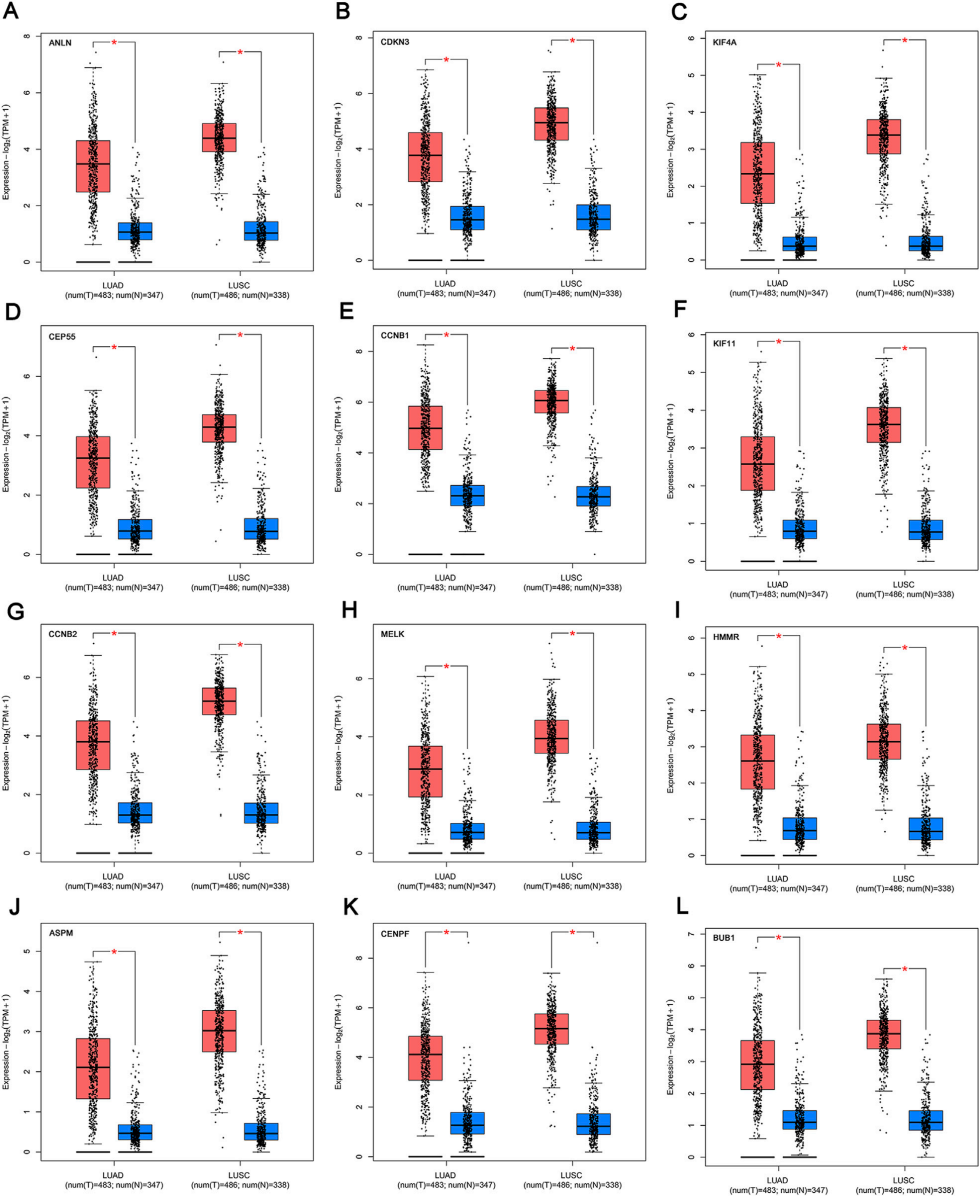
mRNA expression of the key genes **(A‐L)** in NSCLC and normal samples from TCGA and GTEx. *p < 0.01. The red box refers to the tumor group, blue box refers to normal group. NSCLC, non-small cell lung cancer.

We profiled the tissue-wise expression of key genes in NSCLC tissues and normal tissues using GEPIA2. The results revealed that the mRNA expression levels of the 12 key genes in the NSCLC samples were significantly higher than those in normal samples (Figure 5).

### Genetic alteration analysis and key gene-related drugs enrichment analysis

**FIGURE 6:**
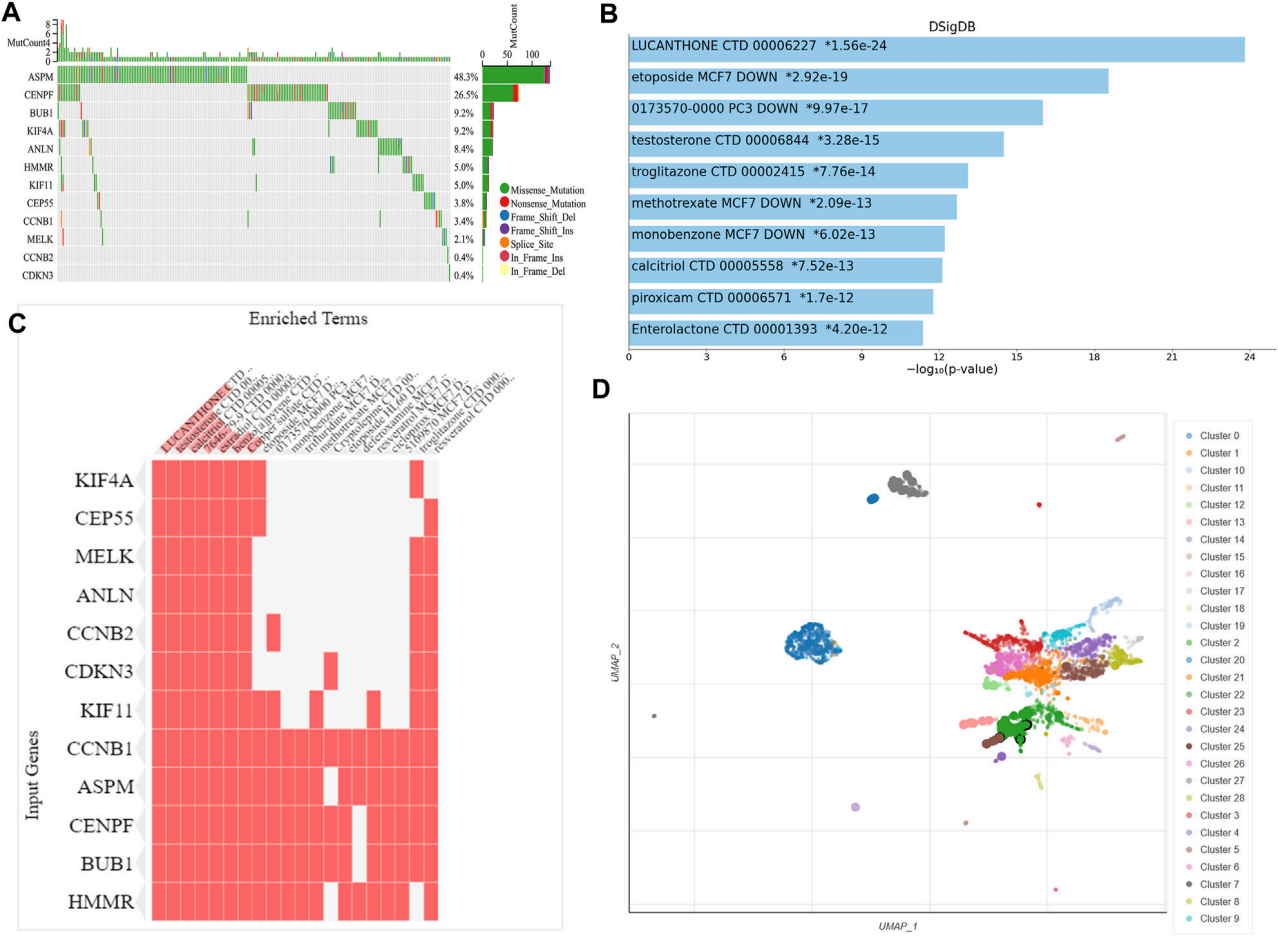
Genetic alteration analysis and enrichment analysis of the key gene-related drugs. **(A)** Mutation profile of the 12 key genes in NSCLC. Enrichment analysis of the key gene-related drugs by Enrichr platform and shown by bar chart **(B)**, heat map **(C)** and scatterplot **(D)**. NSCLC, non-small cell lung cancer.

We observed the mutation status of these key genes in different NSCLC samples of TCGA. As shown in Figure 6A, ASPM had the highest mutation frequency, followed by CENPF. CCNB2 and CDKN3 had the lowest mutation frequency. Missense mutation and frame-shift mutation were the most common types of mutations, while in-frame internal deletion was rare. To explore drugs that associated with the key genes, we used Enrichr platform to perform cluster analysis and UMAP algorithm to draw scatter map for all corresponding terms in DSigDB gene set database. We found that the terms of enrichment of these key genes were correlated with antitumor drugs etoposide and methotrexate, as well as non-tumor drugs such as lucanthone, troglitazone, testosterone, calcitriol and piroxicam (Figure 6).

### Prognostic role of key genes

**FIGURE 7:**
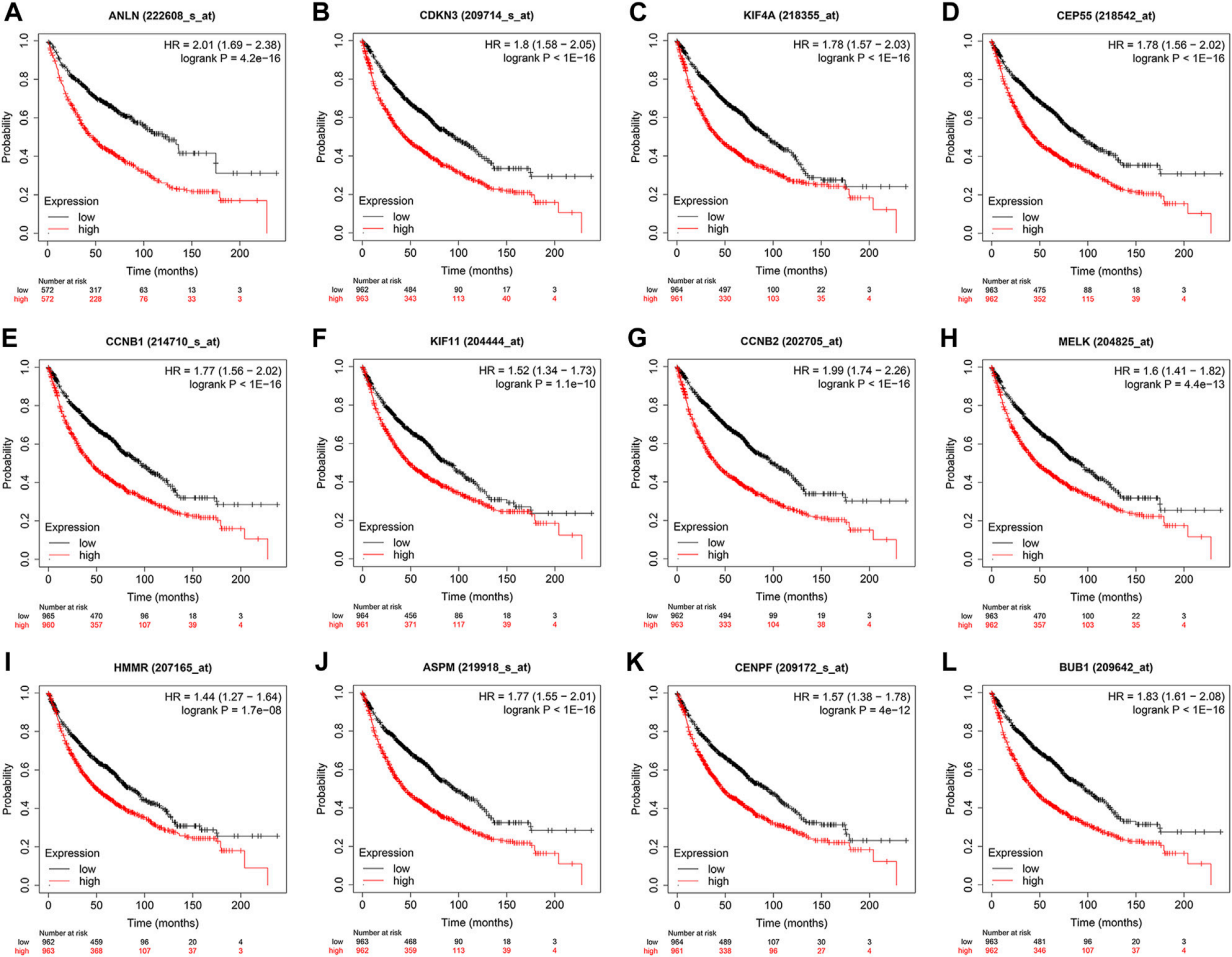
Kaplan–Meier overall survival analyses of the 12 key genes **(A‐L)** in NSCLC patients. NSCLC, non-small cell lung cancer.

**FIGURE 8:**
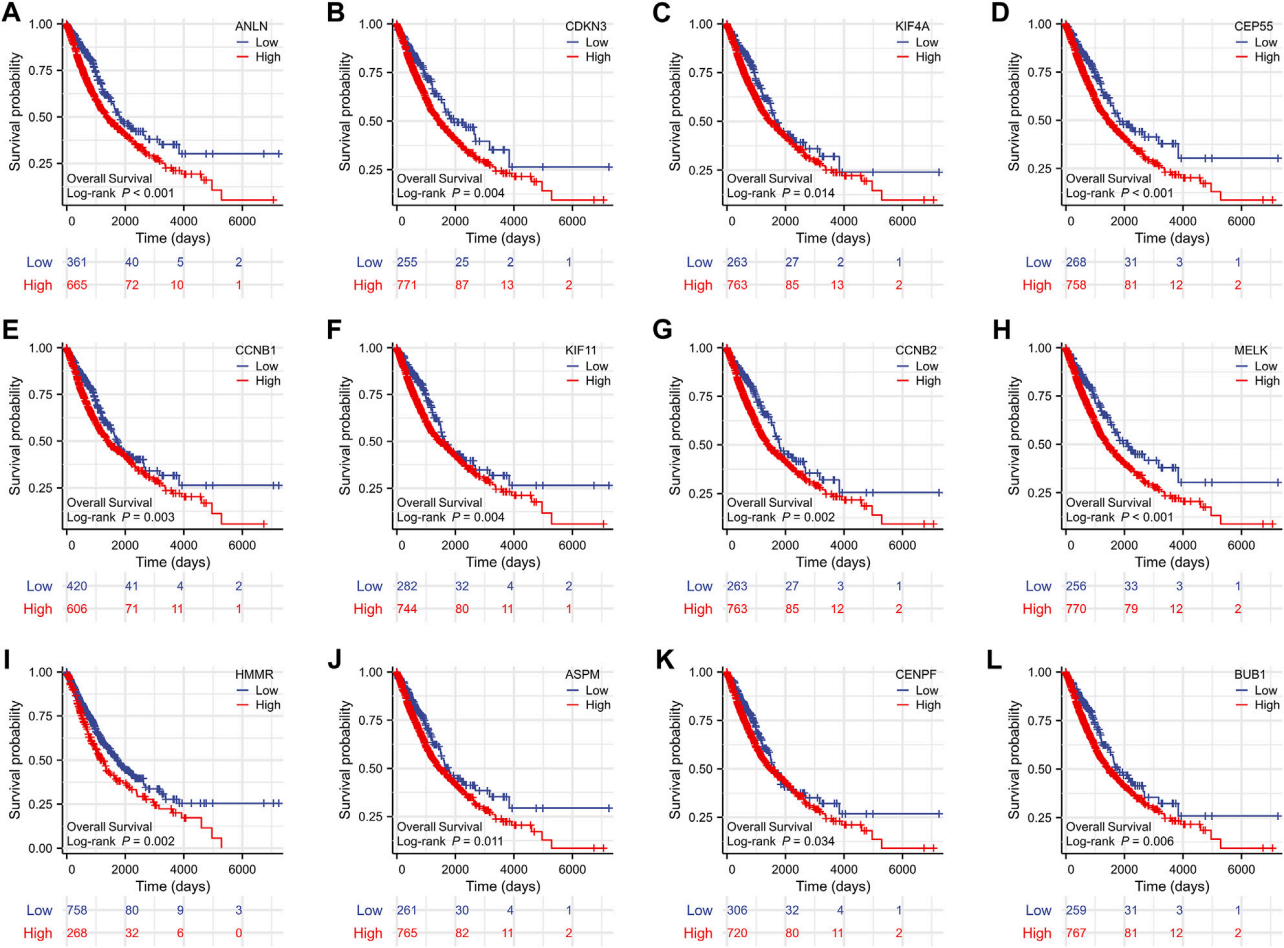
The overall survival analyses of the 12 key genes **(A‐L)** performed by R software using the RNAseq data of NSCLC in the TCGA database. NSCLC, non-small cell lung cancer.

To evaluate the prognostic values of the 12 key genes, we used the Kaplan–Meier plotter, an online database that contained transcriptomic data of 3,452 NSCLC patients. Just as PD-L1 expression, tumor mutational burden can be used to predict immune checkpoint inhibitor outcomes, the key molecules we identified can predict survival outcomes in patients with NSCLC. Overall survival (OS) and first-progression (FP) survival curves are shown in Figure 7; Supplementary Figure S1. High transcriptional levels of the 12 key genes (ANLN, CDKN3, KIF4A, CEP55, CCNB1, KIF11, CCNB2, MELK, HMMR, ASPM, CENPF, and BUB1) were all significantly related to poorer OS (all *p* < 0.001) and FP survival (all *p* < 0.01) in NSCLC. We verified the overall survival analysis of NSCLC patients through the TCGA database, and the conclusion reached was consistent with those obtained by the Kaplan-Meier plotter analysis (Figure 8).

### *In vitro* verification of ANLN and the relationship between its protein expression and the clinicopathologic parameters of NSCLC patients

**FIGURE 9:**
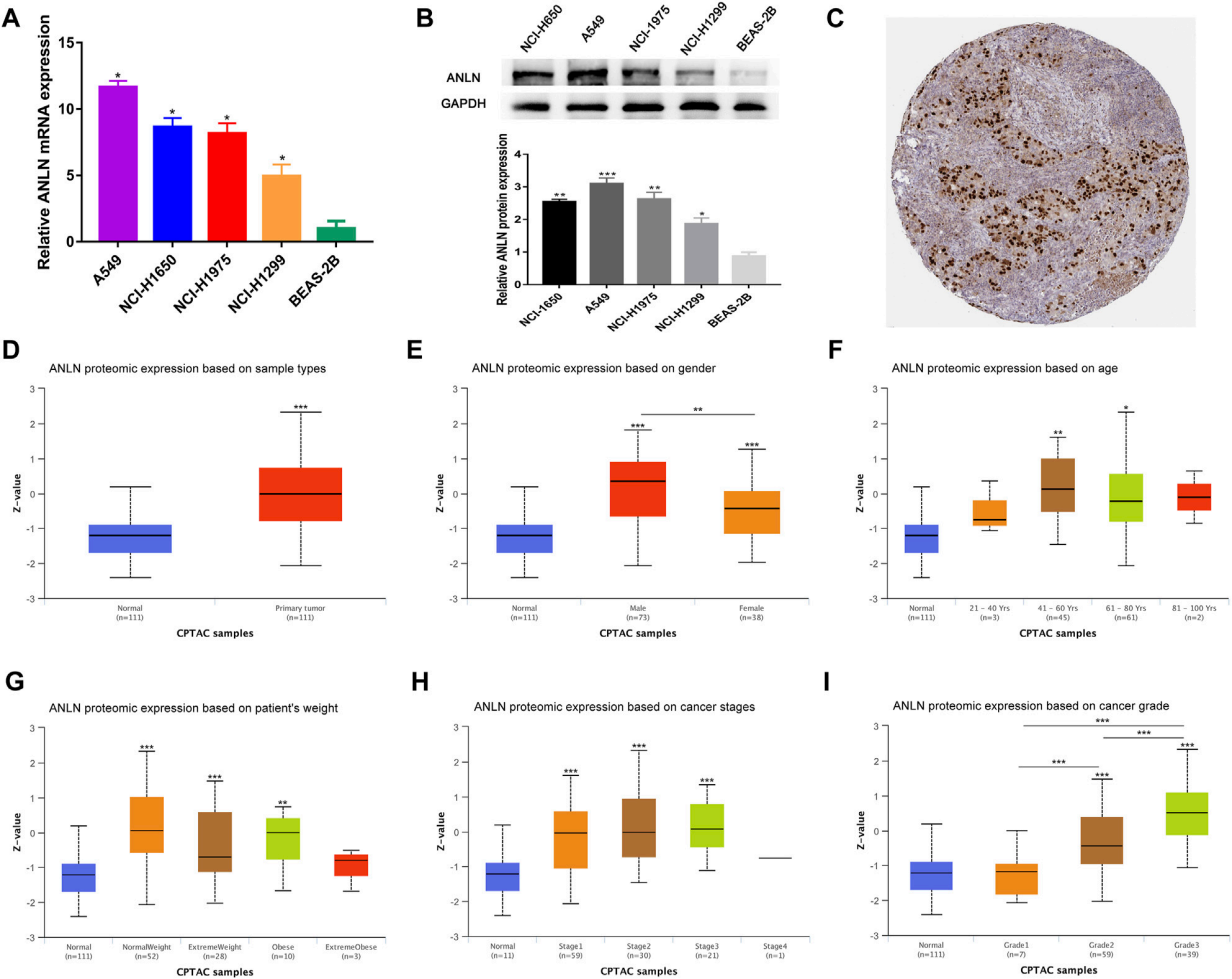
Validation of ANLN mRNA and protein expression and its association with different clinicopathological parameters in NSCLC patients. **(A)** qRT-PCR analysis of ANLN in four NSCLC cell lines and normal lung epithelial cell line. **(B)** Western blotting of ANLN in four NSCLC cell lines and normal lung epithelial cell line. **(C)** Immunohistochemical result of ANLN in lung cancer tissues in HPA database. **(D–I)** Diverse clinicopathological parameters: Sample types, patients' gender, age, weight, pathologic stage and tumor grade. *p < 0.05, **p < 0.01, ***p < 0.001. NSCLC, non-small cell lung cancer.

To verify the transcription level and protein expression level of ANLN, QRT-PCR and Western blotting assays were performed in BEAS-2B and four NSCLC cell lines. We found that both mRNA and protein levels of ANLN in the four NSCLC cell lines were significantly higher than those in BEAS-2B (Figures 9A, B). Through the HPA database, we found that ANLN was strongly positive in the immunohistochemical test of lung cancer tissues (Figure 9C). In addition, we further investigated the relationship of ANLN and various clinicopathological parameters of NSCLC and its gene expression profile in different cancer types. There was a gradually increasing trend based on the protein expression of ANLN from grade I to grade III, while age, weight, and tumor stage groups did not significantly differ given the protein expression of ANLN (Figures 9D–I). Interestingly, we also found that ANLN level of was higher in male patients than in female patients (Figure 9E, *p* < 0.01). As shown in Supplementary Figure S1, ANLN is elevated in various TCGA and GTEx tumors, including hepatocellular carcinoma, pancreatic adenocarcinoma, and breast carcinoma, compared with paired normal tissues.

## Discussion

With rapidly increasing morbidity and mortality, the 5-year survival of lung cancer patients varies from 4% to 17%, depending on the region and stage (Hirsch et al., 2017). Substantial progress has been made in NSCLC treatment in recent years, but long-term effective responses are still rare for most patients (Herbst et al., 2018). It remains critical to explore the underlying pathogenesis of lung cancer and achieve more precise treatment. A number of researchers have made impressive progress in this area, exploring the microenvironment of tumors, looking for biomarkers and individual targeted treatment strategies (Guo et al., 2022; Jiang et al., 2022).

Rather than focusing on a single cohort study, we integrated four cohorts of microarray databases and identified 126 overlapping DEGs (37 upregulated and 89 downregulated) in this study. Through further functional clustering and enrichment analyses, we found that these genes were mainly enriched in the mitotic nuclear division, cell cycle G2/M phase transition, and PPAR signaling pathway. Mitotic nuclear division, a biological process that is complementary to but opposite to apoptosis, plays a crucial part in carcinogenesis, tumor cell maintenance, and tumor progression (Sinha et al., 2019). Given that cancer is a cell cycle disease, the progression of the cell cycle is inextricably linked to the proliferation and activation of cancer cells. The progression of the cell cycle is coordinated by the continuous activation of cyclin-dependent kinases through their corresponding cyclin chaperone (Malumbres, 2014). Some tumor suppressor genes and drug molecules can inhibit tumor cell proliferation and invasion by arresting the cell in the G2/M phase transition (Song et al., 2009). PPARs have three subtypes (PPAR-α, PPAR-β and PPAR-γ), which exhibit diverse roles in vertebrates. PPAR-α mainly plays a role in removing circulating lipids or cell lipids, PPAR-β is involved in lipid oxidation and cell proliferation, while PPAR-γ activation enhances the proliferation of cancer cells and promotes brain metastasis (Bougarne et al., 2018; Magadum and Engel, 2018; Zou et al., 2019). To further explore the internal interactions of the overlapping DEGs, a PPI network was developed. 12 genes with the strongest connectivity in the network were identified. High transcriptional levels of these genes were significantly correlated with poor prognosis, which reveals their potential prognostic value.

ANLN, the top gene in our modules, is a unique scaffolding protein that was first isolated from *Drosophila melanogaster* embryos and was mainly associated with cytokinesis (Zhang and Maddox, 2010).ANLN has been reported to be overexpressed in many tumors. It is involved in the progression of pancreatic, brain, breast, and lung cancers (Hall et al., 2005; Olakowski et al., 2009; Magnusson et al., 2016; Long et al., 2018), which is consistent with our experimental results. Evidence has shown that ANLN promotes cell proliferation, and the loss of ANLN prevents the cancer cells from dividing and reduces their migration and invasion (Wang et al., 2019). Furthermore, there is also evidence showing that ANLN expression correlates with lung adenocarcinoma metastasis (Xu et al., 2019). In breast cancer, ANLN was found to be a alternative marker for Ki-67 (cell proliferation index), which is consistent with our findings (Figure 6F). Based on the evidence supporting the correlation of ANLN with acknowledged features of cancer, ANLN should be considered as a novel target for cancer therapy.

CDKN3 has been reported to be overexpressed in glioma and cervical cancer, and its over-expression is associated with inferior survival (Yu et al., 2007; Espinosa et al., 2013). Since there are more mitotic cells in rapidly proliferating tumor cells, CDKN3 transcription and protein levels fluctuate throughout the cell cycle, reaching a peak during mitosis. High levels of mitotic CDKN3 expression is the most likely mechanism for frequent CDKN3 mRNA over-expression in human cancer (Fan et al., 2015). The cell cycle-dependent elements of CCNB1 and CCNB2 are essential for meiotic resumption. CCNB1 has been observed to expedite tumor cell division, cell proliferation, and tumor growth in colorectal and pancreatic cancers (Fang et al., 2014; Zhang et al., 2018). CCNB2 is also correlated with cancer progression and inferior prognosis in breast cancer, hepatocellular carcinoma and NSCLC (Qian et al., 2015; Li et al., 2019; Jayanthi et al., 2020). KIF4A, the kinesin family member 4A, plays a key role in process of DNA replication and repair. It promotes cell proliferation, correlates with the size of the tumor in oral carcinoma, and serve as a potential prognostic indicator in various solid tumors (Wu et al., 2008; Rouam et al., 2010). KIF11 (E.g.,5) and MELK have been identified as oncogenes in multiple tumors and inhibiting agents targeting them have entered phase I/II clinical trials with encouraging results (Ganguly et al., 2014; Garcia-Saez and Skoufias, 2021). As of now, nine clinical trials targeting KIF11 have been completed, and five clinical trials targeting MELK are ongoing or completed, according to ClinicalTrials.gov (https://clinicaltrials.gov/). These drugs are used alone or in combination with other medicines to treat patients with refractory cancers.

CEP55 was identified as an ideal cancer vaccine candidate (Inoda et al., 2011) and a marker for predicting cancer invasion risk, metastasis, and therapeutic outcome (Tandon and Banerjee, 2020). HMMR, alternatively called RHAMM or CD168, is a microtubule-associated protein that regulates mitosis and meiosis. (Chen et al., 2018). Abnormal expression of HMMR disrupts the microtubule process during cell division and leads to abnormalities in the mitotic spindle, altering the fate of progenitor cells and leading to genomic instability (Pujana et al., 2007). HMMR has been reported to be closely linked to cancer risk and progression in various tumor types (Rein et al., 2003). Currently, there are limited researches on ASPM’s role in tumors. Recently, it has been reported as a new predictor of tumor aggresiveness and prognosis in bladder, prostate, and endometrial cancers. (Pai et al., 2019; Saleh et al., 2020; Zhou et al., 2020). The prenylated protein CENPF has been used clinically as a proliferative marker for malignant tumor cell growth (Varis et al., 2006). BUB1, a serine/threonine-protein kinase, plays a crucial part in oncogenesis, chromosome arrangement, and spindle assembly (Bolanos-Garcia and Blundell, 2011).

Finally, we profiled the tissue-specific expression of key genes in NSCLC and normal specimens from TCGA database and found that its mRNA levels were significantly elevated in tumor than in adjacent non-tumor tissues. We further explored the clinical implication of ANLN, and its protein expression showed a gradually increasing trend from grade I to III, revealing its association with tumor aggressiveness.

## Conclusion

Through multiple microarray datasets and integrated bioinformatics analysis, we identified key genes and pathways that may be involved in NSCLC carcinogenesis, which are mainly associated with mitosis, vasculogenesis, and G2/M transition of the mitotic cell cycle. These findings provide new insights and opportunities for further development of prognostic biomarkers and therapeutic targets for NSCLC patients.

## Data Availability

The original contributions presented in the study are included in the article/Supplementary Material, further inquiries can be directed to the corresponding authors.
