# Supplementary material for: Identification and validation of novel prognostic biomarkers and therapeutic targets for non-small cell lung cancer
Source: Front Genet. 2023 Mar 16;14:1139994. doi: 10.3389/fgene.2023.1139994 (PMC10060803; doi:10.3389/fgene.2023.1139994)
Supplement: Supplementary file 1 [file DataSheet1.ZIP › Supplemental Materials/Supplemental material caption.docx]

**Supplementary materials**

**Figure S1.** First-progression survival curves for the expression of the top 12 hub genes in NSCLC patients.

**Figure S2.** The gene expression profile of ANLN across all TCGA and GTEx tumor and normal samples. Each dots represent expression of samples.

**Table S1.** Top 12 hub genes in STRING interaction network ranked by density of maximum neighborhood component.
