# Supplementary material for: Identification and validation of novel prognostic biomarkers and therapeutic targets for non-small cell lung cancer
Source: Front Genet. 2023 Mar 16;14:1139994. doi: 10.3389/fgene.2023.1139994 (PMC10060803; doi:10.3389/fgene.2023.1139994)
Supplement: Supplementary file 1 [file DataSheet1.ZIP › Supplemental Materials/Table S1.docx]

Table S1. Top 12 hub genes in STRING interaction network ranked by density of maximum neighborhood component

|  | |  | |  | |  | |  | |  | |  | |  |
| --- | --- | --- | --- | --- | --- | --- | --- | --- | --- | --- | --- | --- | --- | --- |
| Rank | Name | | Score | |  | |  | |  | |  | |  | |
| 1 | ANLN | | 1.04155 | |  | |  | |  | |  | |  | |
| 2 | CDKN3 | | 1.04154 | |  | |  | |  | |  | |  | |
| 3 | KIF4A | | 1.04101 | |  | |  | |  | |  | |  | |
| 4 | CEP55 | | 1.04101 | |  | |  | |  | |  | |  | |
| 5 | CCNB1 | | 1.02811 | |  | |  | |  | |  | |  | |
| 6 | KIF11 | | 1.02811 | |  | |  | |  | |  | |  | |
| 7 | CCNB2 | | 1.02811 | |  | |  | |  | |  | |  | |
| 8 | MELK | | 1.02811 | |  | |  | |  | |  | |  | |
| 9 | HMMR | | 1.02811 | |  | |  | |  | |  | |  | |
| 10 | ASPM | | 1.02811 | |  | |  | |  | |  | |  | |
| 11 | CENPF | | 1.02811 | |  | |  | |  | |  | |  | |
| 12 | BUB1 | | 1.02811 | |  | |  | |  | |  | |  | |
