# Supplementary figures and images for: Identification and validation of novel prognostic biomarkers and therapeutic targets for non-small cell lung cancer
Source: Front Genet. 2023 Mar 16;14:1139994. doi: 10.3389/fgene.2023.1139994 (PMC10060803; doi:10.3389/fgene.2023.1139994)

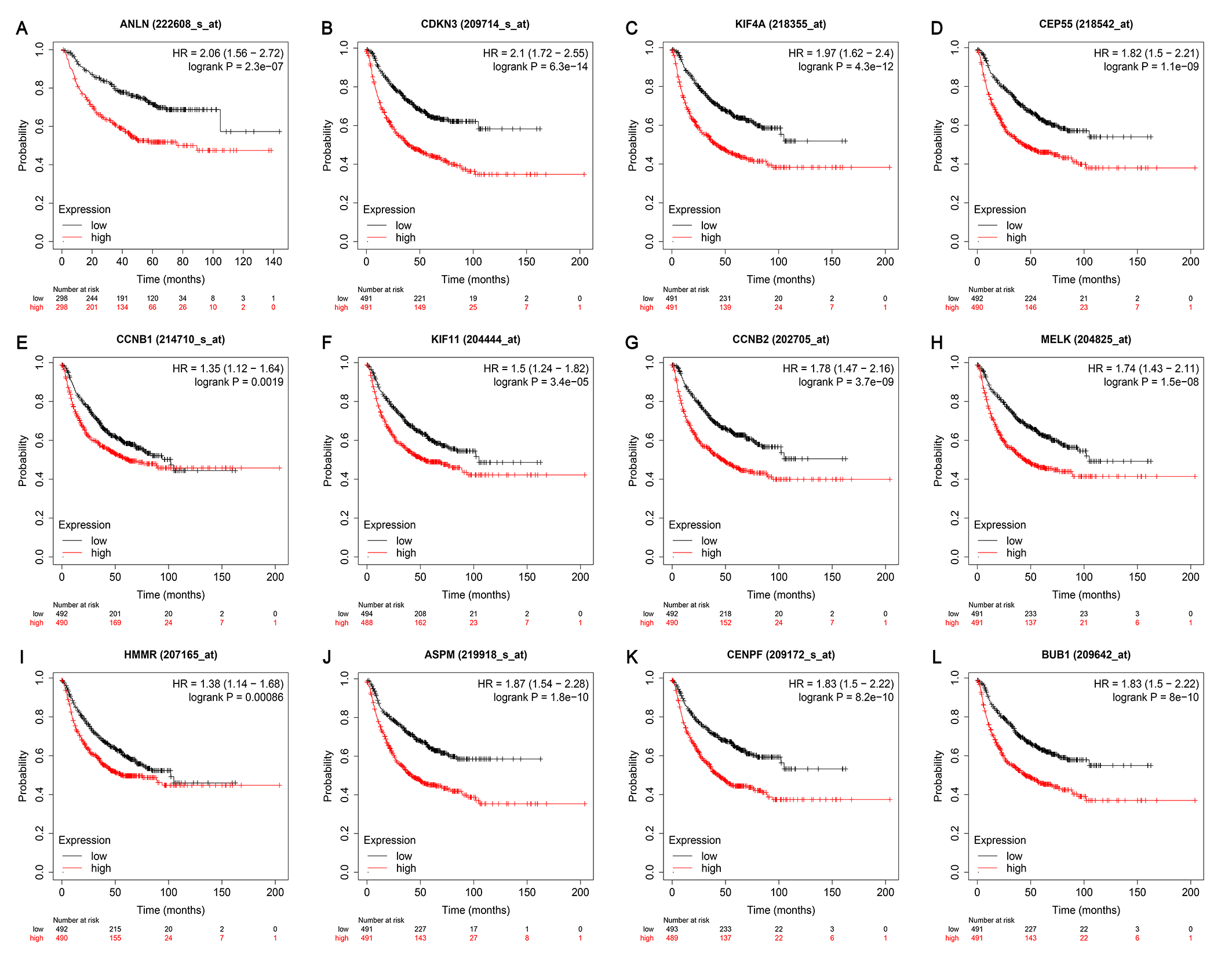

Supplement: Supplementary file 1 [file DataSheet1.ZIP › Supplemental Materials/Figure S1.tif]

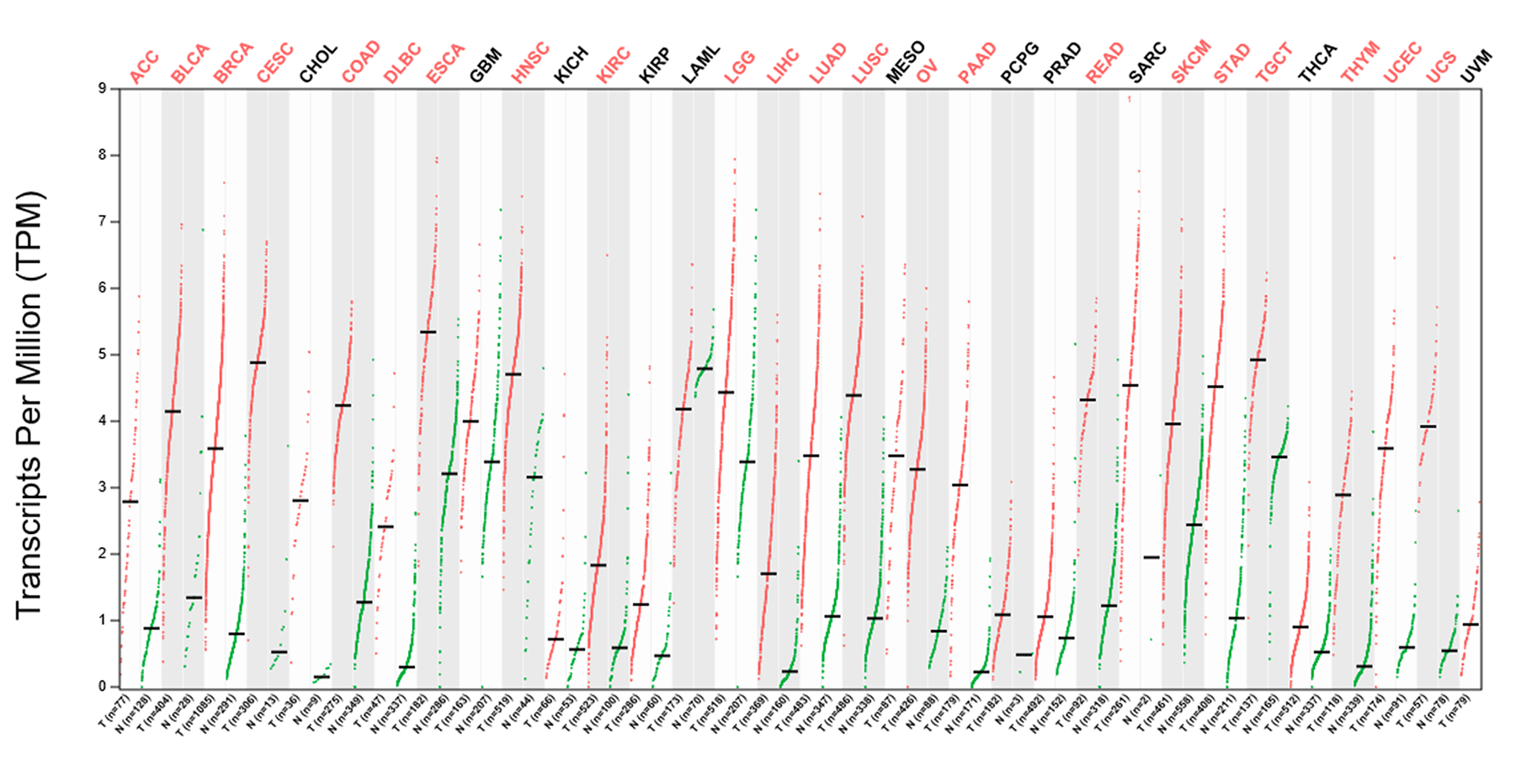

Supplement: Supplementary file 1 [file DataSheet1.ZIP › Supplemental Materials/Figure S2.tif]
